# Optogenetic study of the response interaction among multi-afferent inputs in the barrel cortex of rats

**DOI:** 10.1038/s41598-019-40688-2

**Published:** 2019-03-08

**Authors:** Yueren Liu, Tomokazu Ohshiro, Shigeo Sakuragi, Kyo Koizumi, Hajime Mushiake, Toru Ishizuka, Hiromu Yawo

**Affiliations:** 1 Department of Integrative Life Sciences, Tohoku University Graduate School of Life Sciences, Sendai, 980-8577 Japan; 2 Department of Physiology, Tohoku University Graduate school of Medicine, Sendai, 980-8575 Japan; 3 Present Address: Department of Pharmacology, Yamagata University School of Medicine, Yamagata, 990-9585 Japan

## Abstract

We investigated the relationship between whisker mechanoreceptive inputs and the neural responses to optical stimulation in layer 2/upper 3 (L2/U3) of the barrel cortex using optogenetics since, ideally, we should investigate interactions among inputs with spatiotemporal acuity. Sixteen whisker points of a transgenic rat (W-TChR2V4), that expresses channelrhodopsin 2 (ChR2)-Venus conjugate (ChR2V) in the peripheral nerve endings surrounding the whisker follicles, were respectively connected one-by-one with 16 LED-coupled optical fibres, which illuminated the targets according to a certain pattern in order to evaluate interactions among the inputs in L2/U3. We found that the individual L2/U3 neurons frequently received excitatory inputs from multiple whiskers that were arrayed in a row. Although the interactions among major afferent inputs (*MAI*s) were negligible, negative interactions with the surrounding inputs suggest that the afferent inputs were integrated in the cortical networks to enhance the contrast of an array to its surroundings. With its simplicity, reproducibility and spatiotemporal acuity, the optogenetic approach would provide an alternative way to understand the principles of afferent integration in the cortex and should complement knowledge obtained by experiments using more natural stimulations.

## Introduction

Vertebrate brains and most invertebrate brains consist of huge numbers of neurons that are connected to each other to make complex networks. For example, in a human brain, there are a hundred billion neurons, each of which receives input from hundreds or thousands of synaptic connections. The afferent inputs from sensory organs of various modalities are thus integrated in the neuronal network to generate brain functions that determine the motor outputs. The operational principles of a model neural network have been investigated through analyzing input-output relationships as a kind of multi-dimensional transfer function of time.

Optical stimulation using optogenetics has advantages over other artificial inputs, such as electrical stimulation, because of its high spatiotemporal precision^1–5^, enabling targeted expression of the light-gated ion channels and targeted irradiation with a given spatiotemporal pattern. Recently the circuit specificity involved in the motor behavior^6–10^, the interregional cortical connectivity^11–14^, and the primary visual cortical responses^15^ have been successfully mapped in the brain of a rodent *in vivo* using optogenetics to stimulate cerebral neurons in combination with behavioral outputs, electrophysiological recordings, optical measurements and/or fMRI^16–18^. However, the cortical response is not merely a summation of the input signals. For example, two parallel inputs reciprocally inhibit the cortical response when they arrive within a brief time window. Although optical stimulation is ideal for analyzing the interactions among inputs because of its spatiotemporal acuity, only a limited number of such investigations have been made^19^.

To investigate the interactions among multi-afferent inputs, we analyzed the relationship between whisker mechanoreceptive inputs and the layer 2/upper 3 (L2/U3) responses in the barrel cortex, where the non-linear input-output relationship has been well established^20–22^. Previously, we generated a transgenic rat line (W-TChR2V4) that expresses ChR2-Venus conjugate (ChR2V) in the dorsal root ganglion (DRG) neuron^23,24^. In this rat model, ChR2V was also expressed in the mechanoreceptive neurons in the trigeminal ganglion as well as in their peripheral nerve endings surrounding the whisker follicles^25^. In the present study we connected each of the 16 whisker points of this rat one-by-one with each of 16 LED-coupled optical fibres, which illuminated the targets according to a certain pattern in order to investigate the interactions among inputs in L2/U3. Although individual L2/U3 neurons do not receive inhibitory whisker inputs, negative interactions or disfacilitation were frequently found between the major excitatory inputs and the inputs from their surroundings.

## Results

### Neural responses to the whisker photostimulation

**Figure 1:**
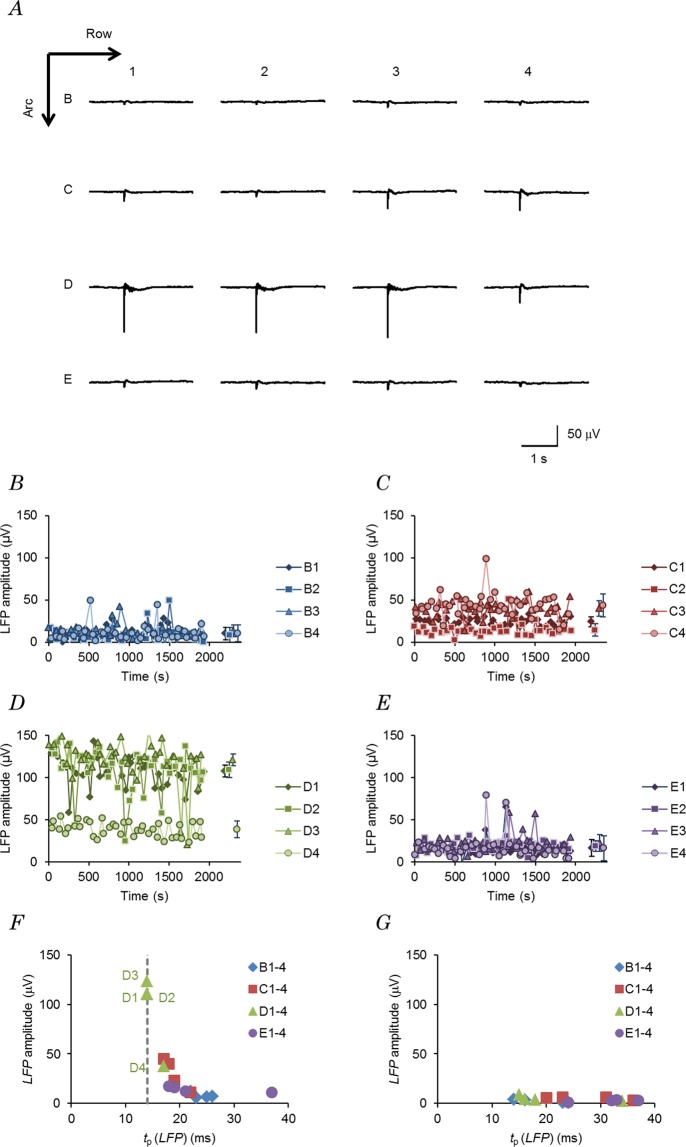
Cortical responses to optogenetic afferent inputs. (**A**) Sample averaged traces of the local field potential (*LFP*) from a fixed point in layer 2/upper 3 (L2/U3) of the S1 barrel cortex in response to the mode-1 photostimulation of the whisker points: B1–4, C1–4, D1–4 and E1–4. Case, 171205U. Each arrow indicates the whisker direction of the row and arc, respectively. (**B**–**E**) The LFP amplitude was plotted as a function of the recording time for the photostimulation at each whisker point: B1–4 (**B**), C1–4 (**C**), D1–4 (**D**) and E1–4 (**E**). The symbol with error bars indicates mean ± SD of the collected data for a series of records. (**F**) The relationship between the *LFP* amplitude and its peak time from the onset of the light pulse. A typical record shown in (**A**). (**G**) Similar to (**F**), but from another off-target experiment in which the end of each optical fibre was set in the centre between 4 adjacent whisker follicles.

While recording at a fixed point in L2/U3 of the S1 barrel cortex, each whisker point was photostimulated one-by-one in a random order (mode 1). Figure 1A shows the sample average records of 40 local field potentials (*LFP*s) in response to each photostimulation at one of the whisker points, B1–4, C1–4, D1–4 and E1–4 (sample 171205U). Although the LFP amplitude varied from one photostimulation to another, those evoked by D1, D2 or D3 photostimulation were robust and could be discriminated from the others throughout this experiment (Fig. 1B–E). The *LFP* by D1, D2 or D3 was also evoked with minimal latency and the time between the onset of photostimulation (*t*_ON_) and the maximal negative peak (time to peak), *t*_p_(*LFP*) was 14 ms (Fig. 1F). On the other hand, the photostimulation at another whisker point evoked a smaller *LFP* with a larger *t*_p_(*LFP*). When the end of each optical fibre was set in the centre between 4 adjacent whisker follicles (off-target experiments), the cortical response to the photostimulation was negligible at every whisker point (Fig. 1G). The maximal LFP of the off-target experiments ranged between 2.4 and 8.4 μV (n = 3 animals) at L2/U3, whereas that of the on-target experiments ranged between 24 and 190 μV (n = 20 animals).

**Figure 2:**
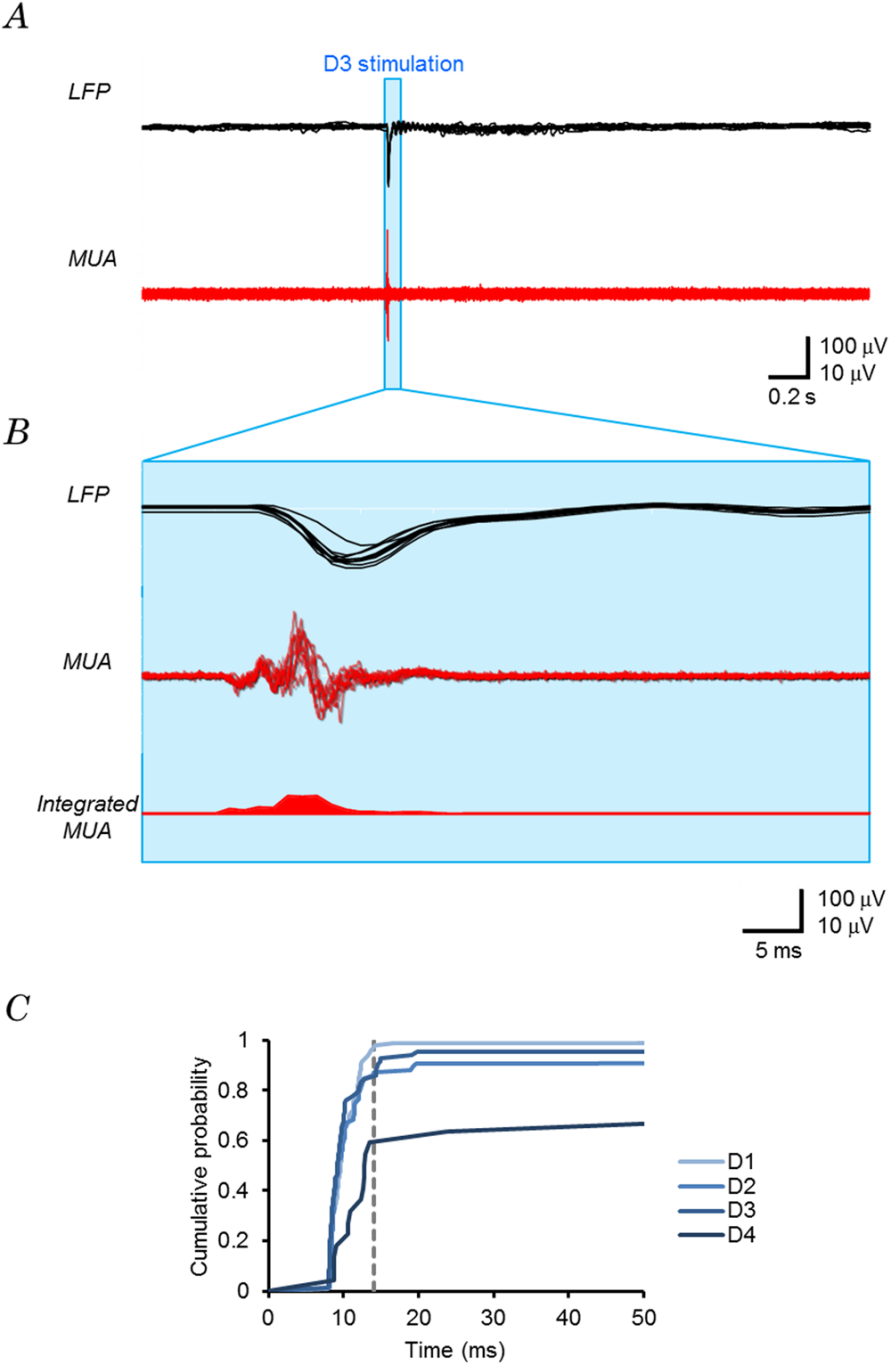
The earliest single unit activity (*eSUA*). (**A**) The *LFP* (top) and the multi-unit activity (*MUA*, bottom) from the experiments, 171205U, shown in Fig. 1A. The D3 whisker point was blue-LED illuminated during the period shown in blue stripe (duration, 50 ms). (**B**) The early responses during illumination period: top, *LFP*; middle, *MUA* and bottom, integrated *MUA* (overlay of 10 traces). (**C**) Cumulative probability plots of the sorted spike timing of the mode-1 responses. The broken line indicates the peak time of the earliest *LFP*.

The photostimulation at these receptive points (D1, D2 and D3) also evoked multi-unit activity (*MUA*) in the neurons with short latency (Fig. 2A). The *MUA* was manifest before the maximal negative peak of *LFP* and was almost silent after 20 ms from *t*_ON_ even when the whisker point was illuminated for 50 ms (Fig. 2B, middle). The absolute *MUA* amplitude was integrated for every 1 ms and plotted against its time after *t*_ON_ (Fig. 2B, bottom). Again, it reached the maximum with a response time less than the *t*_p_(*LFP*) by photostimulation at D1, D2 or D3. The single unit activity (*SUA*) was sorted and counted between −1 and +2 s of *t*_ON_ throughout the experiment (40 photostimulations on 16 whisker spots). A significant fraction of *SUA* was evoked between *t*_ON_ and *t*_p_(*LFP*) (Fig. 2C). Hereafter, we focused on those *SUA* between 0 and 50 ms after *t*_ON_, which we called the earliest *SUA* (*eSUA*).

### Multi-afferent responses

**Figure 3:**
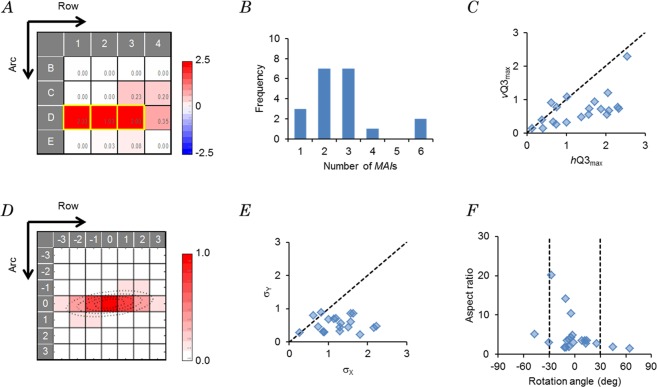
Multi-afferent responses. (**A**) The first regression coefficient values (${\hat{a}}_{i}$) obtained from the *eSUA* data shown in Fig. 2C (case, 171205U) were mapped in a 4 × 4 matrix. The major afferent inputs (*MAI*s) were enclosed in yellow frames. Each arrow indicates the whisker direction of the row and arc, respectively. (**B**) Summary of the *MAI* number. (**C**) Orientation preference of the inputs. The ratio of the maximal horizontal Q3 (*h*Q3_max_) to the maximal vertical Q3 (*v*Q3_max_) (*h*Q3_max_/*v*Q3_max_ ratio) was plotted (n = 20). (**D**) An autocorrelogram made from the first regression coefficient values (${\hat{a}}_{i}$) shown in A (case, 171205U). Four contours of the fit ellipse were drawn at equal intervals and superimposed over the autocorrelogram. Each arrow indicates the whisker direction of row and arc, respectively. (**E**) Summary of the σ_X_/σ_Y_ ratio from a two-dimensional Gaussian function fitted to the autocorrelogram (n = 20). A broken line was drawn at σ_X_/σ_Y_ = 1. (**F**) Summary of the rotation angle (abscissa) and the aspect ratio (ordinate) of the ellipse fitted to the autocorrelogram (n = 20). Broken lines were drawn at the rotation angles of −30 and 30 deg.

To evaluate the response pattern of a neuron for the multi-whisker stimulation, the spike response data from the mode-1 experiment (individual whisker point photostimulation) and that from the mode-2 experiment (simultaneous multi-whisker points photostimulation, see Methods) were combined and regressed to a quadratic polynomial function (eq. 1). Figure 3A shows the first regression coefficient estimates (${\hat{a}}_{i}$, *i* = 1~16) from the *eSUA* data shown in Fig. 2C (171205U) in a 4 × 4 heat map. Here, we defined the major afferent inputs (*MAI*s) as those that evoked a response over 50% of the maximum, the D1, D2 and D3 inputs in the case of Fig. 3A. The spatial response pattern was similarly investigated for every sample, which varied from one to another (Supplementary Fig. S1). Although some ${\hat{a}}_{i}$ had negative signs, their absolute values were negligible as compared to the maximal ${\hat{a}}_{i}$ value. Indeed, the maximum negative/maximum positive ratio ranged 0~7.7% (n = 20). In 17 of 20 samples, the spatial response pattern consisted of multiple *MAI*s (Fig. 3B).

For an *eSUA*, the excitatory whisker inputs were frequently arrayed along a row of whisker points (horizontally to the rat) as exemplified in Fig. 3A. To evaluate the orientation preference of the inputs, the 3rd quartile (Q3) was calculated for each group of four ${\hat{a}}_{i}$ values in the horizontal row or vertical column (arc direction) of the 4 × 4 map made from the *eSUA* data. In the case, 171205U, shown in Fig. 3A, the horizontal Q3 was maximal for the third row of D1-D2-D3-D4 with a value of 2.08, whereas the vertical Q3 was maximal for the third arc of B3-C3-D3-E3, with a value of 0.67. The fact that the maximal horizontal Q3 (*h*Q3_max_) was larger than the maximal vertical Q3 (*v*Q3_max_) indicated a preference for the horizontal orientation. As shown in Fig. 3C, the *h*Q3_max_ and the *v*Q3_max_ were calculated for every sample and compared. We found an input preference predominantly in the row direction (15/20 samples) with statistical significance (*p* < 0.005, Wilcoxon signed rank test).

The global features of the spatial response pattern could also be characterized by curve-fitting. However, the direct fitting to a 4 × 4 response pattern map such as that shown in Fig. 3A would not reliable because of the low spatial resolution. On the other hand, the 7 × 7 autocorrelogram computed from the first regression coefficient estimates (${\hat{a}}_{i}$) of the *eSUA* data (Fig. 3D) increased the resolution without affecting the spatial frequency spectrum (Wiener-Khinchin theorem), and was well fitted by ellipsoid contours (bivariate Gaussian function, see Methods). The coefficient of the determination value (*r*^2^) of this example, 171205U, was 0.99, indicating that the fitting was excellent. Similar results were obtained for the other records (Supplementary Fig. S2). For every sample, the σ_X_ and the σ_Y_ were calculated and compared. As summarized in Fig. 3E, the responsive pattern was more directed to the row than to the arc with statistical significance (*p* < 0.00005, n = 20, Wilcoxon signed rank test). As shown in the distribution of the shape parameters, aspect ratio (σ_X_′/σ_Y_′) and rotation angle, the major axis of the elongated ellipse was preferentially oriented horizontally (Fig. 3F). Indeed, the orientation angle was between −30 and 30° in 16/20 samples. These characteristics of the autocorrelogram were consistent with the notion that the response pattern of the whisker photostimulation was preferentially arrayed in the row direction.

### Interaction among afferent inputs

**Figure 4:**
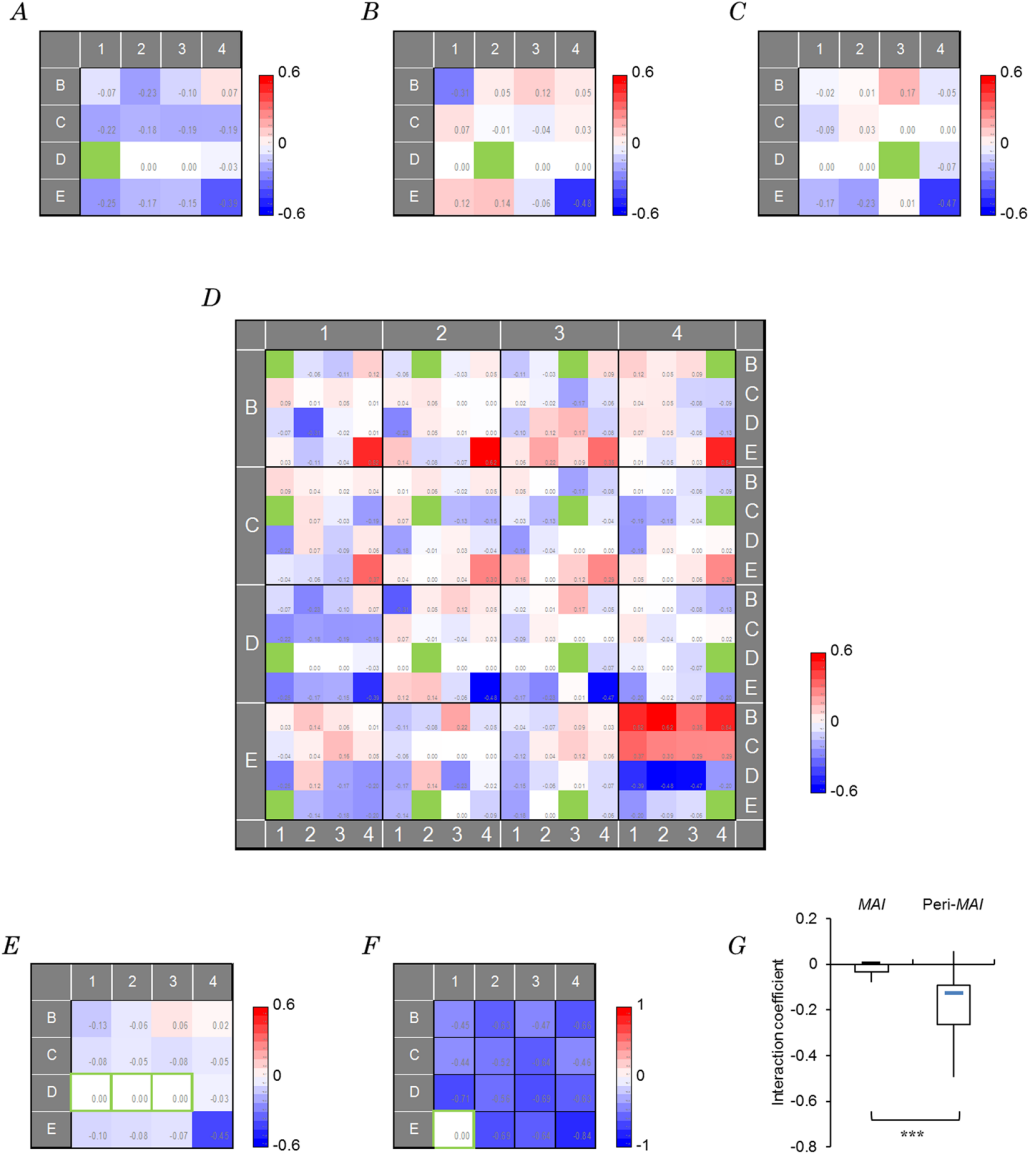
Interaction among afferent inputs. (**A**–**C**) The interaction between each whisker photostimulation and the reference input (a green-filled cell) at D1 (A), D2 (**B**) and D3 (**C**) was respectively represented as 4 × 4 maps of interaction coefficients (*c*_*jk*_) obtained from the *eSUA* data shown in Fig. 3A (case, 171205U). (**D**) The assembled (4 × 4)^2^ map of interaction coefficients (*c*_*jk*_). (**E**) The ensemble 4 × 4 interaction map made for *MAI*s consisting of D1, D2 and D3, the cells of which are encircled in green. (**F**) Similar to E but from another sample which had a single *MAI* at E1 (case, 171106U). (**G**) Box-and whisker plot summary of the average interaction coefficients among *MAI*s and among the inputs beside *MAI* (peri-*MAI*) of the ensemble 4 × 4 interaction map. ***, *p* < 0.005, Wilcoxon signed rank test (n = 17).

Are the multiple afferent inputs interacting to drive a neuron in the cortex? Figure 4A shows a sample 4 × 4 map of the interaction coefficients (*c*_*jk*_) with reference to the D1 photostimulation from the *eSUA* data (171205U) shown in Fig. 3A (see Methods). At a glance, the D1 input had negative interaction with B1–3, C1–4, D4 and E1–4 inputs. However, the interaction with D2 and D3 was negligible. On the other hand, the D2 input showed positive interaction with B2–3, C1, C4 and E1–2, although it had negligible interaction with D1 and D3 (Fig. 4B). As shown in Fig. 4C, the D3 input again showed negligible interaction with D1 and D2, whereas its negative interaction with D4, E1 and E2 was obvious. Similar 4 × 4 maps of interaction coefficients were made with every whisker photostimulation and assembled to form a (4 × 4)^2^ interaction map (Fig. 4D). As shown in the ensemble 4 × 4 interaction map made for *MAI*s consisting of D1, D2 and D3 (Fig. 4E), the *MAI*s were surrounded by the negatively interacting inputs (peri-*MAI*s), which contained C1–3, D4, and E1–4, whereas the negative interaction was negligible among *MAI*s. Similarly, the (4 × 4)^2^ interaction maps were obtained for 19 other records (Supplementary Fig. S3).

Among the 20 recordings of *eSUA*, 17 showed at least 2 *MAI*s but 3 had a single *MAI*. Figure 4F shows a 4 × 4 interaction map with the E1 input from the *eSUA* data of another sample (171116U), which had a single *MAI* at E1. In this case, the interaction between this *MAI* and the other was always negative. Similarly, ensemble 4 × 4 interaction maps for *MAI*s were obtained for 18 other records (Supplementary Fig. S4). Mostly there was negligible interaction among the *MAI*s (11/17 samples), whereas there was negative average interaction among the *MAI*s in 6/17 samples. When the average interaction between each *MAI* and the non-*MAI*s surrounding it (peri-*MAI*s) was calculated, it was positive in 2/17 samples and negative in 15/17 samples. As shown in Fig. 4G, the average interaction between the *MAI* group and peri-*MAI* group showed significantly larger negativity than that within the *MAI* group inputs (*p* < 0.005, n = 17, Wilcoxon signed rank test).

## Discussion

In the present study the afferent inputs in a neuron were mapped in the S1 barrel cortex L2/U3 of W-TChR2V4 rats that expressed ChR2 in the mechanoreceptive neurons in the TG. Although there was some dependency on the intensity of the photostimulation, the activation of the ChR2 photocurrent was generally very fast and reached its peak within several ms^1,2^. When the membrane potential is depolarized, the latency to evoke an action potential is also determined by the membrane properties of the neuron. Indeed, a single action potential was evoked in 1–10 ms in the ChR2-expressing TG neurons from the W-TChR2V4 rats^25^. As a result, the optogenetic inputs on the whisker area arrived at the somatosensory cortex synchronously within 20 ms from their onset. This optical characterization of the input-response relationship in S1 as a function of time should be robust because using the same rat line, Go-task conditioning was established and recalled by the whisker area irradiation as a function of the power and duration of the light pulse^26,27^. Indeed, the *LFP* amplitude was robust by the *MAI*s throughout every mode 1 experiment and specifically distinct from those by the peri-*MAI*s (Supplementary Fig. S5). Since ChR2 is exclusively expressed in the large mechanoreceptive neurons in the TG^25^, the photostimulation is presumed not to be accompanied by the sense of pain, which would produce unpredictable effects on the cortical computation. The likelihood of the optical stimulation evoking other non-specific inputs such as those arising from the intervibrissal fur/skin, from passing nerves and from the eyes appears to be negligible based on the cortical response to the control off-target photostimulation. We also noted that the cortical response was absent when the fibre end was not exactly above the whisker follicle.

As every whisker point, B1–4, C1–4, D1–4 and E1–4, was respectively connected to a fibre-coupled LED, the output of which was pulse-controlled independently, any spatiotemporal pattern of stimulation could be given with the highest precision. The spatial response pattern of afferent inputs was thus mapped for *eSUA* using two modes. Mode 1, in which the whisker spot was photostimulated individually, is suitable for evaluating the number and contribution of individual inputs. On the other hand, mode 2, in which a combination of 4 whisker points out of 16 was photostimulated at once, is suitable for analyzing interactions, facilitative or suppressive, among inputs.

Anatomically, three kinds of afferent fibres innervate around the whisker follicle sinus complex (FSC)^28,29^. The superficial vibrissal nerves (SVNs) innervate the superficial layers with Merkel endings or with dense circumferentially oriented lanceolate endings. On the other hand, a single deep vibrissal nerve (DVN) sends its branches to the middle-to-deep layers of the FSC to form neural plexuses with various forms of endings. At the base of FSC, thin-caliber fibres penetrate to form a meshwork. All of these mechanoreceptive afferents were expressing ChR2 in our W-TChR2V4 rat^25^. However, as a result of tissue absorption and scattering, the visible light is strongly attenuated with distance^30,31^: blue light (473 nm) was estimated to become <1% at a 1-mm depth in the brain^32^. The light penetration was directly measured using depilated rat skin and was only 3.7 ± 0.6% (n = 6). Therefore, in our experiments, since the LED light hardly activated the DVN terminals, the response map likely represents only the surface layer afferents around FSC.

Accumulating evidence supports the notion that a significant number of the supragranular layer neurons in the layer 2~deep 3 were activated by the deflection of more than one whisker, in contrast to the L4 barrel neurons, which are mainly responsive to the deflection of only one whisker^33^. This appears to be one of the specific traits of the supragranular layer neurons in which the simultaneous or successive stimulation of two whiskers in a row preferentially facilitates the neuronal activity^34–36^. The whole cell recording from a supragranular layer neuron also revealed that the subthreshold receptive field was elongated along rows within 10–150 ms following a deflection^37^. Consistent with these previous studies that used the mechanical deflection of whiskers, our optical stimulation suggested that the L2/U3 neurons often receive multiple *MAI*s that are preferentially arrayed horizontally to the animal (row direction) as evidenced by the distribution of the *h*Q3_max_/*v*Q3_max_ ratio and rotation angle of the autocorrelogram of ${\hat{a}}_{i}$. That is, the inputs from a row of whiskers such as D1–4 are divided by the inputs from the adjacent rows such as C1–4 and E1–4. Infrequently, we sampled a response pattern that consisted of a single *MAI* or multiple non-adjacent *MAI*s in the 4 × 4 map of the ${\hat{a}}_{i}$ (eg. cases 171031U and 171116U, Supplementary Fig. S1). Probably, the responsive pattern of a L2/U3 neuron is dependent on the recording site in the barrel field. Although every cortical response was recorded at a single fixed point in the S1 barrel cortex (see Methods), its position relative to the barrel structure as well as its depth likely varies among individual rats. Indeed, the supragranular layer neurons that are preferentially responsive to the simultaneous (correlated) stimuli of multiple whiskers are distributed at the periphery of each barrel to the septal region and may receive thalamic inputs from the VPM head^38^, whereas those that were responsive to the uncorrelated stimuli were frequently distributed above the centre of the corresponding barrel due to VPM core inputs^37^. However, the response pattern in the deep L3 should be studied in the future because of its unique connectivity with other layers^39,40^.

One remarkable feature of the interaction between afferent inputs revealed in this study was the non-linearity in the response summation. Although the negativity of ${\hat{a}}_{i}$ was negligible in every response map, a sub-additive summation was typically observed in the response to the multiple stimuli applied on the *MAI*s. Such an interaction may be accounted for by a ceiling effect of the neuronal response, in which one of the *MAI*s is strong enough to evoke a maximally attainable response of the neuron. Therefore, another nearby input would gain few additional responses. A physiological limitation of the neural membrane in its excitability due to, for instance, a finite number of ion channels present on the cell surface, may trivially account for this ceiling effect. However, a network-mediated interaction among the inputs is a more likely mechanism. In this study, we showed clear suppressions of the *MAI* response by additional stimulation at whiskers that were ineffective when presented alone (Fig. 4). Since the additional stimulus was seemingly ineffective, a simple ceiling effect could not explain the decrease in the response.

In the present study the interaction between afferent inputs was confirmed by mapping the interaction coefficients (*c*_*jk*_) for *eSUA*. As noted previously, multiple whiskers often formed a zone consisting of inputs of large weights (${\hat{a}}_{i}$). Although the inputs in this zone interact negligibly or weakly with each other, they often interact negatively with other inputs distributed around the zone. Probably, the afferent inputs from the zone were sculptured by the surrounding whisker inputs before arriving at the L2/U3 neuron (disfacilitation) to enhance the sensitivity of a horizontally arrayed whisker row against the background^22,41^. Indeed, the activity of regular spiking neurons in the L3 and L4 was inhibited by vibration of the adjacent whiskers in a manner dependent on its number, whereas that of the thalamocortical inputs was not^42^. The input from a single whisker was suppressed by the preceding deflection of adjacent whiskers without inhibiting the postsynaptic response^43^. While the non-linear interaction was prevalently suppressive at any frequency of whisker deflection (0.5–8 Hz) among neurons through the layers in the barrel cortex, it was facilitatory at 8 Hz between whiskers along an arc in the L5/6 neurons^44^. Although, in the present study, four whiskers were photostimulated simultaneously using a 50 ms light pulse at low frequency (0.33 Hz), their interaction should be investigated further in the future as a function of the time interval between inputs^45^.

Although artificial, the results of our optogenetic mapping of multi-whisker inputs are consistent with those of previous studies using mechanical deflection of the whiskers^22^. However, several disadvantages, such as the absence of directional information of whisker deflection^33^ and the depth of light penetration, have to be overcome in the future. On the other hand, with its simplicity, reproducibility, specificity and spatiotemporal acuity, the optogenetic approach would still be suitable for revealing principles of afferent integration in the cortex, especially with the expected technical improvements in the future, and should complement knowledge obtained by experiments using more natural stimulations. To the best of our knowledge, this is the first study that evaluated the orientation preference of multi-whisker inputs quantitatively. These new analytical methods as well as the interaction coefficient mapping could be applied to other data to reveal the logic underlying cortical computation.

## Methods

### Animals

All experiments were carried out using heterozygous offspring of one of the thy1.2 promotor-ChR2-Venus transgenic rat lines, W-TChR2V4 (*W*-*Tg*(Thy1-COP4/YFP*)4Jfhy, NBRP No: 0685, the National BioResource Project - Rat, Kyoto, Japan) with the genetic background of the Wistar strain^23^. The data were collected from 20 rats, both male and female adults (7–12 weeks old, 180–240 gBW). All animal experiments were approved by the Tohoku University Committee for Animal Experiments (Approval No. 2017LsA-001) and were carried out in accordance with the Guidelines for Animal Experiments and Related Activities of Tohoku University as well as the guiding principles of the Physiological Society of Japan and the National Institutes of Health (NIH), USA. The number of animals in this study was kept to a minimum and, when possible, all animals were anesthetized to minimize their suffering. Animals had access to food and water ad libitum and were kept under a 12-hour light-dark cycle.

After immobilizing the rat with isoflurane inhalation, α-chloralose (dose, 0.04 g/kgBW, Sigma-Aldrich Co., St. Louis, MO) was intraperitoneally injected to induce anaesthesia. The anaesthesia was maintained throughout the experiment by intraperitoneal supply of the reagent (1/5 of the initial dose/hr) using a syringe pump (TOP-5500, Top corporation, Tokyo, Japan).

**Figure 5:**
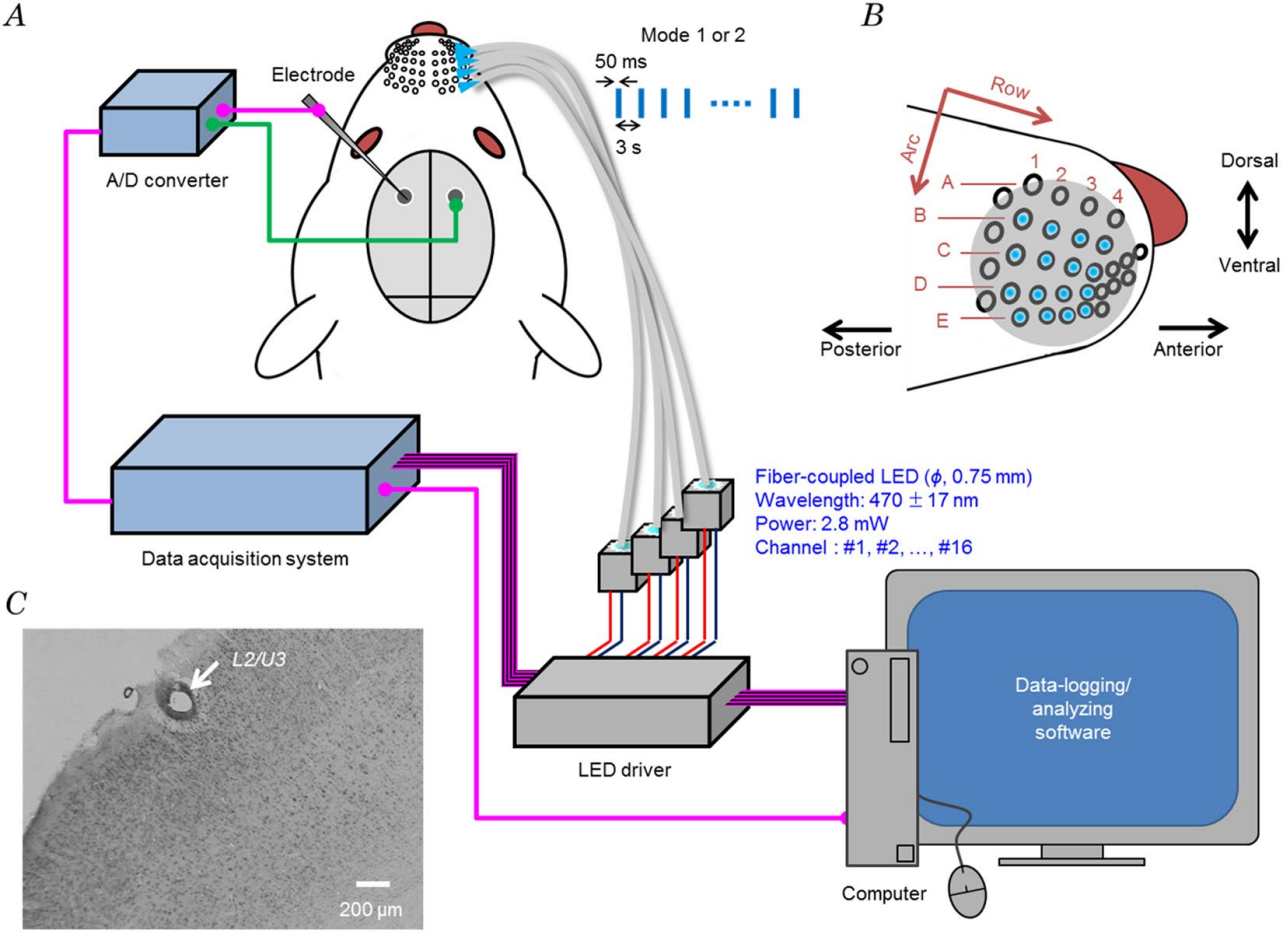
Optogenetic multiple whisker stimulation system. (**A**) Whisker points were connected with 16 LEDs, which were on and off according to the mode-1 or -2 pattern, while the electrical response was recorded from a fixed point in the contralateral S1 barrel field. (**B**) Photostimulated whisker points: B1–4, C1–4, D1–4 and E1–4. Each arrow indicates the whisker direction of row and arc respectively. (**C**) Position of the electrode tip (arrow) in the layer 2/upper 3 (L2/U3) of S1 barrel cortex (Nissl staining).

Once under anaesthesia, the rat was put on an insulation board to maintain its body temperature and all whiskers were trimmed off on the right side using an electric razor. Then, the vibrissal fur was removed using depilatory cream so that the blue light for photostimulation could reach the trigeminal nerve endings around the whisker follicles. After whisker trimming and fur removal, the positions of 30 whisker follicles termed as α, β, γ, δ, A1–4, B1–4, C1–5, D1–6 and E1–7 were marked on a piece of clear mending tape (Scotch^TM^ tape, 3 M Corporate, St. Paul, Minnesota, USA) adhered on the snout using a marker pen. Among them, 16 points corresponding to the whisker addresses (w-ad) B1–4, C1–4, D1–4 and E1–4, were used to match 16 fibre-coupled LEDs regulated by a 16-channel LED driver (Fig. 5A,B). An acrylic sheet (5 mm thick) was attached with the marked tape and a perforation (diameter, 1.0 mm) was made at every mark. A circular coverslip was paced on the acrylic sheet to fabricate the whisker board. The coverslip-side of the whisker board was glued on the snout so that each perforation matched with each whisker follicle. In the case of control off-target experiments, each fibre end was set in the centre between 4 adjacent whisker points. Therefore, the distance between an irradiated point and any whisker points was no more than 1.0 mm.

The fully anaesthetized rat was placed in a stereotaxic apparatus with ear and tooth bars to fix its head. The skull was exposed and cleaned, and the position of the left S1 barrel cortex was marked at *l* × 3.3/9.0 mm lateral from the midline and *l* × 5.0/9.0 mm caudal from the bregma while adjusting the length according to the bregma-lambda distance (*l*)^46^. Then, two pits (diameter, about 2–4 mm) were drilled on the skull; one at the marked position for the recording electrode and another on the right side for the reference electrode. After penetrating the left pit to make the cortical surface visible, the dura mater was carefully removed using a needle.

### Optics

Sixteen fibre-coupled LEDs (470 nm, FCS-0470-000, Mightex Systems, Toronto, Canada) were connected to the 16-channel LED driver (SLC-CA16-U, Mightex Systems) while the individual optical fibres (diameter, 0.75 mm) were inserted in the whisker board in order; LED#1–4 to w-ad B1–4, LED#5–8 to w-ad C1–4, LED#9–12 to w-ad D1–4 and LED#13–16 to w-ad E1–4. The radiant flux was 2.8 mW at the distal end of each optical fibre.

The on/off patterns of the individual LEDs were regulated independently by home-made software. While setting the duration and amplitude at 50 ms and 500 mA, the driving pulses were applied following either one of the 2 modes of photostimulation protocol. Mode 1: each LED was pulse-driven one-by-one in a random sequence with an interval of 3 s during one cycle, and this cycle was repeated 40 times. Mode 2: every combination of four LEDs out of 16 was pulse-driven at once while the combination was made one-by-one in a random sequence with an interval of 3 s. In total, _16_C_4_ (=1820) combinations were made in a series of experiments. Therefore, in total, 16 + 1820 = 1837 spatially different patterns of optogenetic stimulations were applied while recording from a single recording site.

### Electrophysiology

The neuronal activity was recorded by a single pole tungsten electrode insulated by parylene C (0.5~1 MΩ, shank diameter 170 µm, tip diameter 24 µm, O-WELL Co., Tokyo, Japan) attached to an OmniPlex D Neural Data Acquisition System (Plexon Inc., Dallas, TX, USA). The timing signal from 16 channels of the LED driver was recorded by the PlexControl through the external analog input (AI) channels. Under the threshold scanning protocol of the PlexControl software at a gain of ×2000, the unit activities of neurons were monitored with a speaker while inserting the electrode in the L2/U3 (depth: 150~300 μm) of the S1 barrel cortex. The raw voltage signal was band-pass filtered at 40–400 Hz for the local field potential (*LFP*) and 0.4–4 kHz (wide band) for the multi-unit activities (*MUA*s). We typically made an electrical lesion mark after the recording by injecting current (total 400 μC) into the recording electrode to verify the depth of the recording (Fig. 5C). We identified the lesion site subsequently by serial sections (40 μm) of the cortex under Nissl staining.

Once stable spiking activities were obtained, the AIs for stimulus timings, the *LFP*s, the *MUA*s, the wave bursts (WBs), the signals for start and stop timings were recorded with the application of the mode-1 or -2 photostimulation protocols. A mode-1 experiment took around 32 minutes and a mode-2 experiment, around 92 minutes. These data were saved on a hard disk for off-line analysis.

### Data analysis

The *MUA* data were sorted off-line by the manufacturer’s software (Plexon Inc.) to extract the single-unit activities (*SUA*s, spikes) under an appropriate algorithm using principal component analysis of the waveform. Using the NeuroExplorer software (Nex Technologies, Littleton, MA) and the home-made software (MATLAB^®^, The MathWorks, Inc., Natick, MA), each spike timing was logged in relation to the start-stop and photostimulation timings and served for further analyses using the home-made software (C++, Visual Studio, Microsoft Co., Redmond, WA; MATLAB^®^, The MathWorks, Inc.), R (https://www.r-project.org/) and Microsoft Excel (Microsoft Co.).

A neural response to an optogenetic stimulus was typically seen after 10–20 ms from the onset. The neural response after a stimulus was thus sampled between 0 and +50 ms from the stimulus onset, the average baseline activity between −50 and 0 ms was subtracted and the stimulus-related neural response was obtained. The subtraction was done separately for the mode-1 and mode-2 recordings.

To map the spatial response pattern, multiple regression analysis was conducted with the response *R*^*t*^, evoked by the *t-*th stimulus ${s}_{i}^{t}$ (*t* = 1 to 1837). ${s}_{i}^{t}$ was expressed as a stimulus pattern vector whose *i*-th element indicates the presence or absence of a photostimulation at the *i*-th whisker: *i* = 1–4 for B1–4, 5–8 for C1–4, 9–12 for D1–4 and 13–16 for E1–4. The presence of a stimulus was valued as 1, otherwise 0, in the corresponding element. For instance, if stimuli were presented at w-ads B1 (*i = *1), C2 (*i = *6), D2 (*i* = 10) and E3 (*i* = 15), the corresponding stimulus pattern vector will be [1 0 0 0 0 1 0 0 0 1 0 0 0 0 1 0]. These experimental data {${s}_{i}^{t}$, *R*^*t*^} (*t* = 1 to 1837) were then fitted to a quadratic polynomial function:

1 $$R={\sum }_{i}^{16}({a}_{i}{s}_{i})+{\sum }_{j\ne k}^{16}({b}_{jk}{s}_{j}{s}_{k}).$$

The first regression coefficient, *a*_*i*_ (*i* = 1, 2, …, 16) and the second regression coefficient, *b*_*jk*_ (*j* = 1, 2, …, 16; *k* = 1, 2, …, 16) were each uniquely determined using a least-squares fitting algorithm. Hereafter, the least squared error solution of each coefficient is expressed as ${\hat{a}}_{i}$ (*i* = 1, 2, …, 16) or ${\hat{b}}_{jk}$ (*j* = 1, 2, …, 16; *k* = 1, 2, …, 16).

The ${\hat{a}}_{i}$ indicates how much of the activity is gained when the *i-*th whisker is stimulated. Similarly, ${\hat{R}}_{i,j}={\hat{a}}_{i}+{\hat{a}}_{j}+{\hat{b}}_{ij}$ is the expected response when *i-*th and *j*-th whiskers are simultaneously stimulated. Note that ${\hat{b}}_{ij}$ indicates how much the multi-whisker response deviated from the simple sum of the two single whisker responses (${\hat{a}}_{i}+{\hat{a}}_{j}$). The set of the parameter ${\hat{a}}_{i}$ (*i* = 1 to 16) was arranged in a 4 × 4 matrix to map the spatial response pattern.

The interaction between the *i*-th and *j*-th inputs can be evaluated by comparing the multiple whisker stimulation response, ${\hat{R}}_{jk}$ to the simple sum of single-whisker stimulation responses, ${\hat{R}}_{j}={\hat{a}}_{j}$and ${\hat{R}}_{k}={\hat{a}}_{k}$. Here, we defined the interaction coefficient, ${c}_{jk}\,\,$in three cases separately in relation to the sum of ${\hat{R}}_{j}$ and ${\hat{R}}_{k}$, $({\hat{R}}_{j}+{\hat{R}}_{k})$ and the larger value between of ${\hat{R}}_{j}$ and ${\hat{R}}_{k}$, $max({\hat{R}}_{j}+{\hat{R}}_{k})$:

2 $${c}_{jk}={\hat{R}}_{jk}-\,{\rm{\max }}({\hat{R}}_{j}+{\hat{R}}_{k}),\,when\,{\hat{R}}_{jk} < max({\hat{R}}_{j}+{\hat{R}}_{k})[{\rm{Case}}\,1]$$

3 $$=\,{\hat{R}}_{jk}-({\hat{R}}_{j}+{\hat{R}}_{k}),\,when\,{\hat{R}}_{jk} > ({\hat{R}}_{j}+{\hat{R}}_{k})\,[{\rm{Case}}\,2]$$

4 $$=\,0,\,else.[{\rm{Case}}\,3]$$

Case 1 indicates a response suppression in which the best single response was reduced by adding the second stimulus. Case 2 indicates a synergistic response enhancement by two inputs. Case 3 would indicate a ceiling effect in which the multiple whisker response was slightly over the single best response because the single best response was already closed to the maximum attainable response of the neuron. That is, in reference to a given whisker input *J*, a 4 × 4 map of *c*_*Jk*_ (*k* = 1~16) was obtained (e.g. Fig. 4A–C). In this representation, the box filled in green corresponds to the reference whisker position, while other boxes were indicated by the color-rating scale according to the interaction coefficient value relative to the absolute maximum (red: positive, blue: negative) between the reference whisker and the corresponding whisker. For example, the green box at D1 and a blue box at the C1 whisker position suggest a suppressive interaction between two inputs. Similarly, a red box indicates that the activity evoked by the simultaneous stimulation of both whiskers was greater than that expected from the simple sum of the two activities evoked by the stimulation of each whisker independently. These interaction maps were then compiled to form a (4 × 4)^2^ interaction map of every *c*_*Jk*_ (*J* = 1~16, *k* = 1~16) (eg. Fig. 4D). That is, the 4 × 4 *c*_*Jk*_ maps in reference to B1, B2, B3 and B4 were arrayed in the first row, those in reference to C1, C2, C3 and C4 were arrayed in the second row, those in reference to D1, D2, D3 and D4 were arrayed in the third row, and those in reference to E1, E2, E3 and E4 were arrayed in the fourth row. An ensemble 4 × 4 map of the interaction coefficients was also made for the multiple whisker inputs of interest (*J*_1_, *J*_2_, …) by averaging the value in each cell of the 4 × 4 map of *c*_*Jk*_.

The autocorrelogram of a spatial response pattern (eg. Fig. 3D) was fit to ellipsoid contours (bivariate Gaussian function):

5 $$A(X,Y)=\,\frac{c}{2\pi |{\rm{\Sigma }}|\,}\,\exp (-\frac{1}{2}\,[(X-{X}_{0},Y-{Y}_{0}){{\rm{\Sigma }}}^{-1}{(X-{X}_{0},Y-{Y}_{0})}^{T}]),$$

where *X*_0_, *Y*_0_ are the centre of the ellipse, and Σ is a positive-semidefinite symmetric matrix:

6 $${\rm{\Sigma }}=(\begin{array}{cc}{\sigma }_{X}^{2} & {\sigma }_{XY}\\ {\sigma }_{XY} & {\sigma }_{Y}^{2}\end{array})$$

In this fitting analysis, c, *X*_0_, *Y*_0_ and Σ were free parameters to be determined by minimizing the sum of squared errors between the Gaussian function and the autocorrelogram.

We then performed spectral decomposition of the obtained parameter matrix:

7 $$\hat{{\rm{\Sigma }}}=R\ast \hat{{\rm{\Sigma }}}^{\prime} \ast {R}^{T}=rotation(\theta )\ast (\begin{array}{cc}{\sigma }_{X^{\prime} }^{2} & 0\\ 0 & {\sigma }_{{Y}^{\text{'}}}^{2}\end{array})\ast rotation(\,-\,\theta ),\,({\sigma }_{X^{\prime} }\ge {\sigma }_{Y^{\prime} })$$

where, *rotation*(*θ*) is a rotation matrix that transforms the Cartesian coordinate (*X*, *Y*) into a new Cartesian coordinate (*X*′, *Y*′), rotated by angle *θ*. After this rotation, the fit ellipse can be expressed in a simpler form:

8 $$\hat{{\rm{A}}}(\hat{X},\hat{Y})=\frac{\hat{c}}{2\pi |\hat{{\rm{\Sigma }}}|\,}\,\exp (-\frac{1}{2}\,[{(\frac{X^{\prime} -{X^{\prime} }_{0}}{{\sigma }_{X^{\prime} }})}^{2}\,+{(\frac{Y^{\prime} -{Y^{\prime} }_{0}}{{\sigma }_{Y^{\prime} }})}^{2}\,]).$$

Equation (8) indicates that the major axis of the new ellipse is now horizontal. Thus, the angle *θ* required for this rotation is equivalent to the tilt angle of the original ellipse before rotation. *θ* takes a positive value for the counter-clock wise rotation in this analysis. Similarly, the ratio ${\sigma }_{X^{\prime} }:{\sigma }_{Y^{\prime} }$ is the aspect ratio of the ellipse fit to the original autocorrelation function.

All data in the text and figures are expressed as the mean ± SEM and evaluated using the Mann-Whitney *U*-test for the unpaired data, the Wilcoxon signed rank test for the paired data and the one-way Kruskal-Wallis test by ranks for the multi-group data to determine statistical significance, unless stated otherwise. It was judged as statistically insignificant when *p* > 0.05. Regression and curve-fitting analyses were performed using custom-made programs written in MATLAB^®^.

### Ethical approval and informed consent

All animal experiments were approved by the Tohoku University Committee for Animal Experiments (Approval No. 2017LsA-001) and were carried out in accordance with the Guidelines for Animal Experiments and Related Activities of Tohoku University as well as the guiding principles of the Physiological Society of Japan and the National Institutes of Health (NIH), USA. There is no experiment involving humans or human tissue samples.

## Supplementary information

Supplementary figures

## Data Availability

The authors declare that any materials, data and associated protocols in this research will be promptly available to readers without undue qualifications in material transfer agreements.
