# Supplementary material for: Optogenetic study of the response interaction among multi-afferent inputs in the barrel cortex of rats
Source: Sci Rep. 2019 Mar 8;9:3917. doi: 10.1038/s41598-019-40688-2 (PMC6408464; doi:10.1038/s41598-019-40688-2)
Supplement: Supplementary file 1 — Supplementary figures [file 41598_2019_40688_MOESM1_ESM.pdf]

Supplementary Figures

Optogenetic study of the response interaction among multi-afferent inputs in the barrel cortex of rats

Yueren Liu[1], Tomokazu Ohshiro[2], Shigeo Sakuragi[1†], Kyo Koizumi[1], Hajime Mushiake[2], Toru Ishizuka[1], Hiromu Yawo[1*]

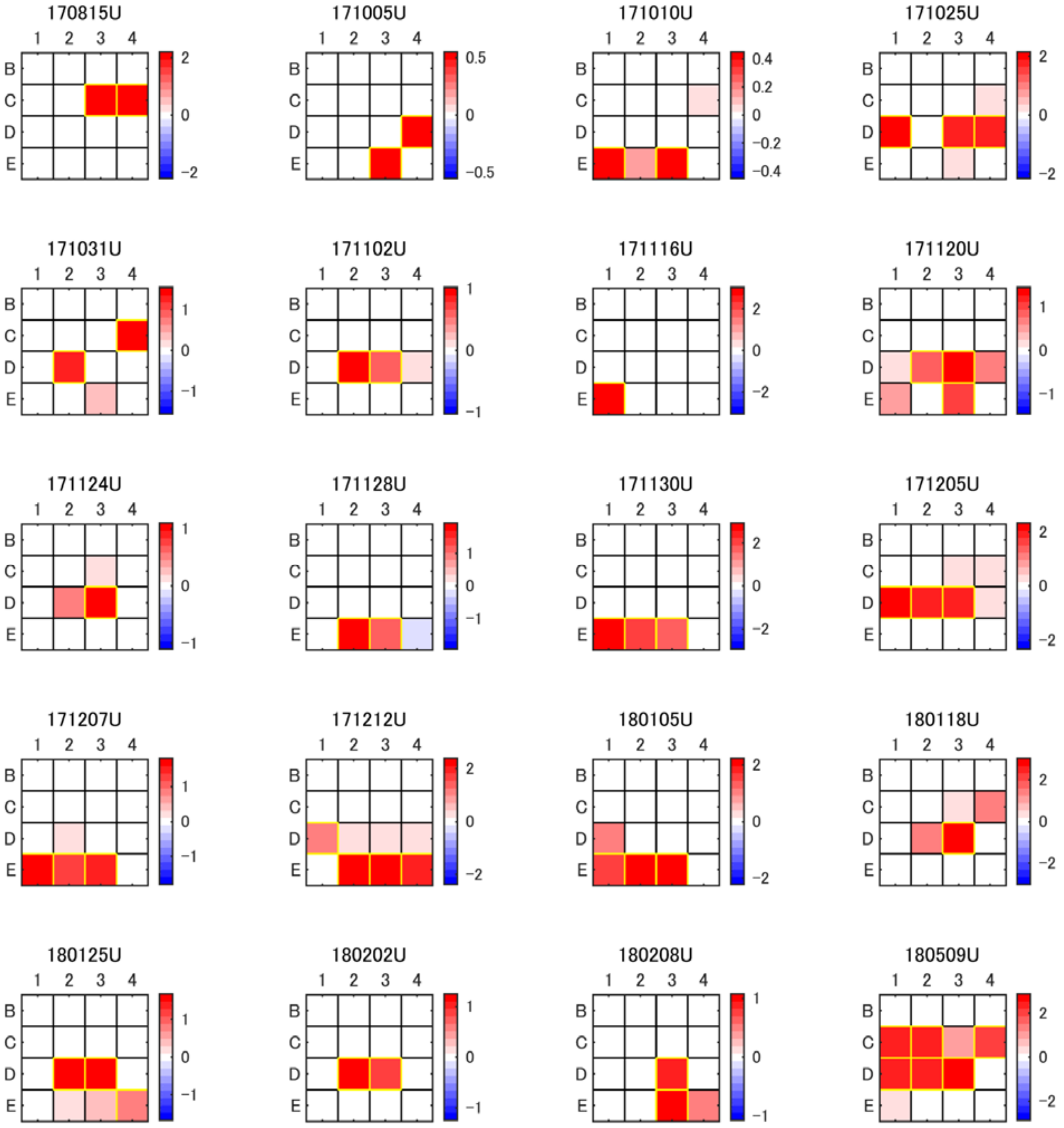

Supplementary figure S1. Each represents a 4 × 4 matrix mapping the first regression coefficients values ($\hat{a}_i$) obtained from each *eSUA* data (n = 20). The major afferent inputs (*MAI*s) are enclosed in yellow frames.

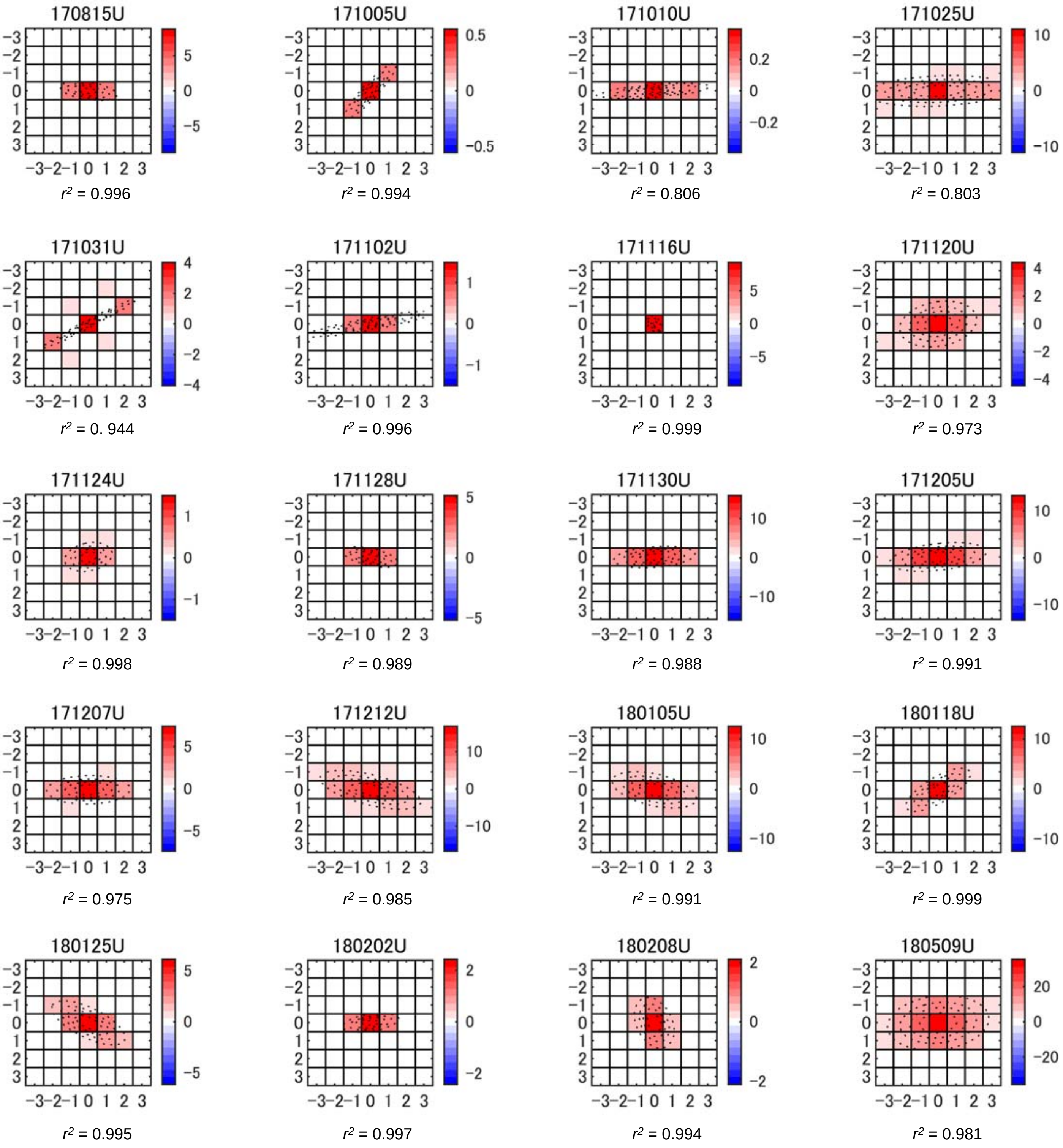

Supplementary figure S2. The autocorrelogram made from a 4 × 4 heat map of the first regression coefficients values ($\hat{a}_i$) from every *eSUA* data (n = 20). Four contours of the fit ellipse were drawn at equal intervals, and superimposed over the autocorrelogram. r^2, coefficient of determination.

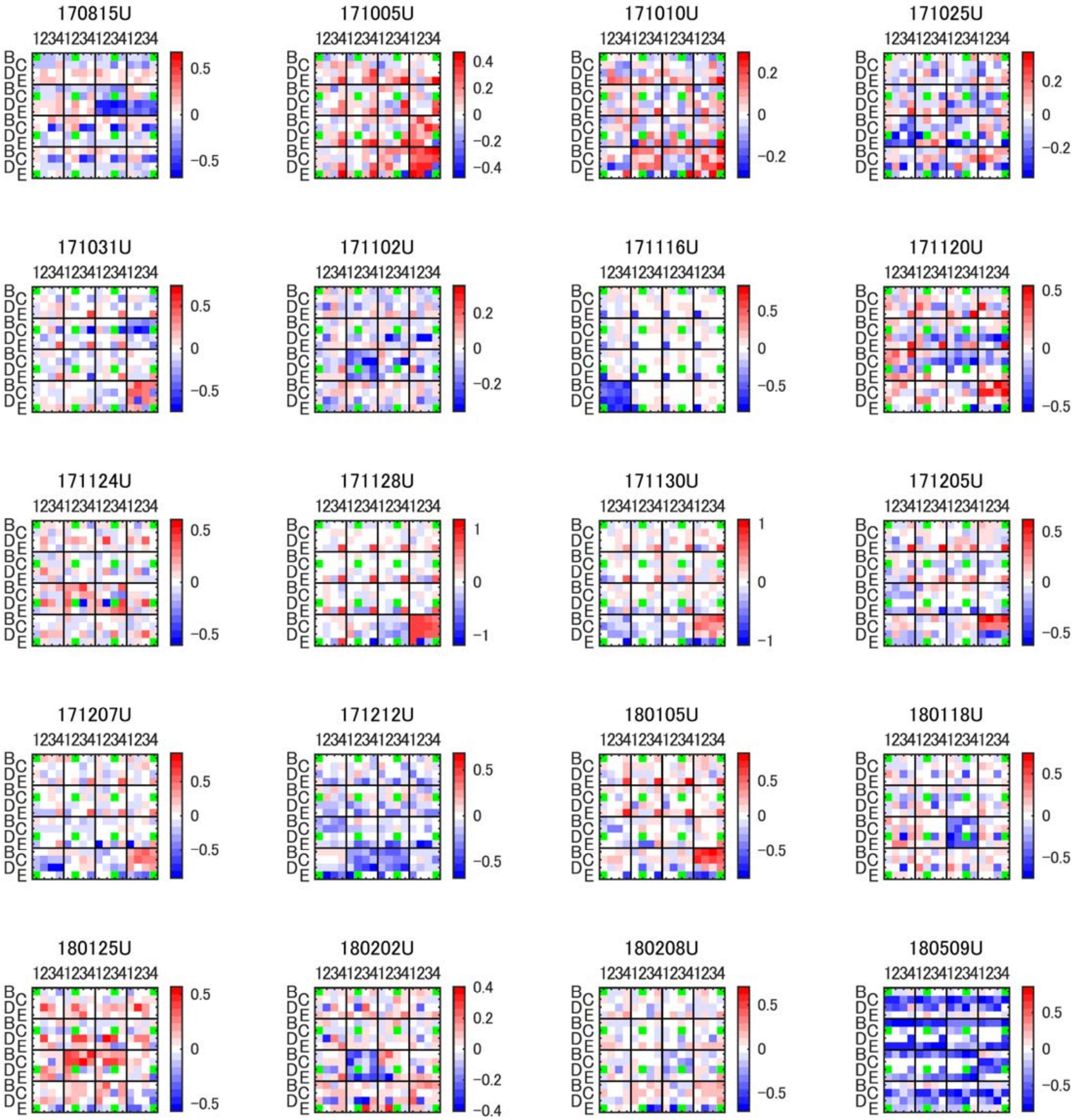

Supplementary figure S3. The assembled $(4 \times 4)^2$ map of interaction coefficients (c_{jk}) was made from every *eSUA* data (n = 20). In each 4 × 4 map, the reference input was green-filled in the cell.

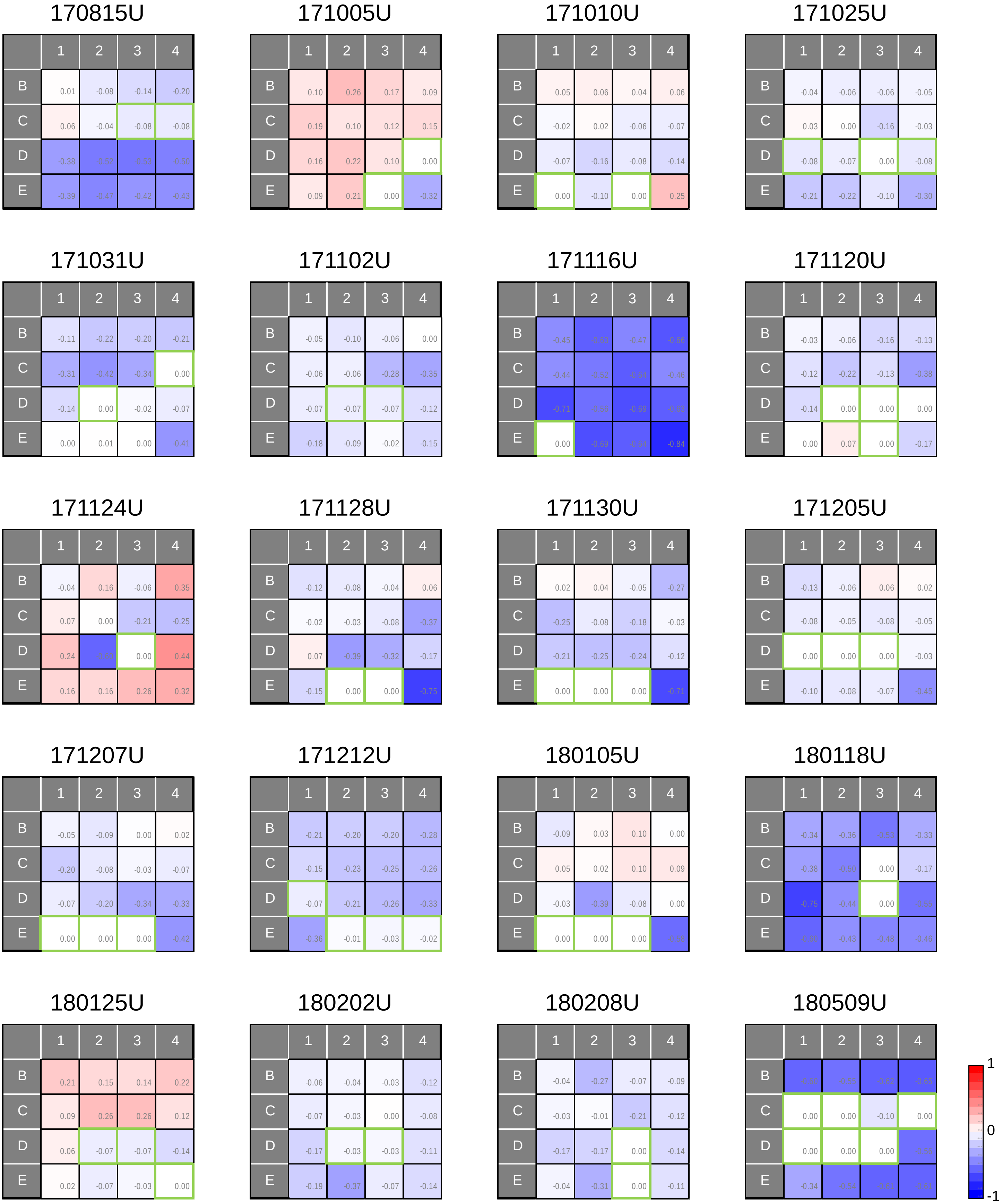

Supplementary figure S4. The ensemble 4 × 4 interaction maps for *MAI*s were made from every *eSUA* data (n = 20). The *MAI*s were encircled in green. The same color-rating scale was applied for all.

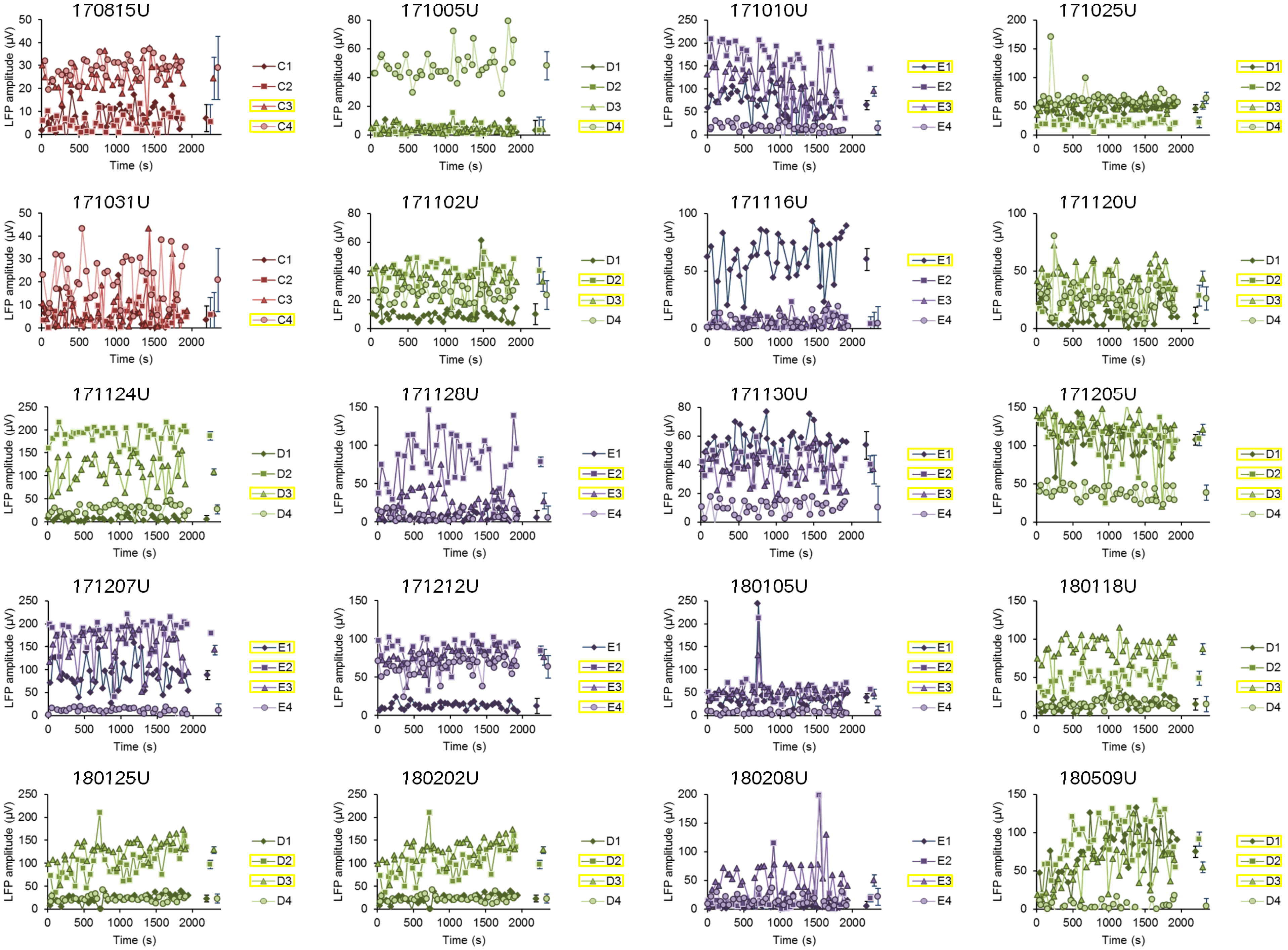

Supplementary figure S5. Robustness and specificity of photostimulation. The LFP amplitude was plotted as a function of the recording time for the photostimulations of each of 4 whiskers in a row containing the most responsive one. The symbol with error bars indicates mean ± SD of the collected data for a record. The MAIs were enclosed in yellow frames.
